# Mechanical and Structural Consequences of PLA/PBAT Contamination in Polypropylene and Polystyrene Recycling

**DOI:** 10.3390/ma18245523

**Published:** 2025-12-09

**Authors:** Przemysław Postawa, Tomasz Stachowiak, Krystyna Malińska, Danuta Dróżdż, Tomasz Jaruga, Arkadiusz Kloziński

**Affiliations:** 1 Department of Technology and Automation, Faculty of Mechanical Engineering, Czestochowa University of Technology, Armii Krajowej 21 St., 42-201 Czestochowa, Poland; tomasz.jaruga@pcz.pl; 2 Department of Sanitary Networks and Installations, Faculty of Infrastructure and Environment, Czestochowa University of Technology, Armii Krajowej 21 St., 42-201 Czestochowa, Poland; krystyna.malinska@pcz.pl (K.M.); danuta.drozdz@pcz.pl (D.D.); 3 Institute of Chemical Technology and Engineering, Poznan University of Technology, Berdychowo 4 St., 60-965 Poznan, Poland; arkadiusz.klozinski@put.poznan.pl

**Keywords:** polymer mixtures, biodegradable polymers contamination, polymer testing, DMTA, impact strength, hardness, tensile strength

## Abstract

The increasing use of biodegradable plastics derived from renewable sources (PLA, PHB, PBAT, and others) in the packaging industry raises controversies and risks related to potentially integrating these plastics into municipal waste streams, which may significantly hinder future recycling efforts. This publication addresses this issue by investigating a selected bio-based and biodegradable commercial mixture of poly(lactic acid) and poly(butylene adipate terephthalate) (PLA/PBAT), referred to as (BIO), in blends with polypropylene (PP) and polystyrene (PS). The blends were prepared with three different mass contents of 1, 5, and 10 wt.% using (PP) and (PS) as base materials. The effects of introducing biodegradable and bio-based plastics into municipal waste streams (PCR—Post-Consumer Recycling), which typically contain polypropylene, various grades of polyethylene, and polystyrene, remain unknown. The purpose of the study was to assess the consequences of contaminating municipal waste destined for recycling (using PP and PS as examples) with small amounts (between 1 and 10%) of BIO plastics. The designed experiment and the obtained results simulate the expected presence of BIO contamination in future PP and PS recyclates. The prepared mixtures were subjected to injection molding to produce test specimens, which were then analyzed for changes in their physical properties such as tensile strength, impact strength and hardness. Thermal properties were assessed using Differential Scanning Calorimetry (DSC), while dynamic properties were analyzed at variable temperatures using Dynamic Mechanical Thermal Analysis (DMTA). The tests provided insights into how the addition of selected, but insignificant ratios (of 1 to 10%) of biopolymers affects the properties of (PP) and (PS) compared to materials without content of biopolymers. The conducted tests of mechanical properties (static and dynamic) and thermomechanical properties have shown that the change in the properties of the mixture depends not only on the amount of biodegradable polymer but also on the nature of the load. It would be advisable to analyze mechanical properties in relation to the duration of the load; therefore, a long-term load analysis is necessary. For dynamic tests, a decrease in impact strength was demonstrated with increasing biodegradable polymer content in the produced mixtures. Similar behavior was recorded for hardness measurements. The results underscore the need for continued research, given the valuable findings for processors and the advancement of mechanical recycling technologies.

## 1. Introduction

Recently, there has been a significant increase in the share of thermoplastics, either bio-based or produced using bacterial processes, in global and European manufacturing. Statistical data highlight the growing potential of these materials in select industries, particularly in the packaging, agriculture, and medical sectors [1,2]. Biodegradable polymers offer a solution to the increasing waste management challenges associated with the end-of-life cycle of petroleum-based polymer products. Despite the rising interest from processors and consumers in applications and products made from biodegradable polymers, and their increasing market share, there are still no legal regulations or clear legislative positions on the separation or management of the problem of mixing two incompatible waste streams: bio-based polymers and petroleum-based polymers [1,2].

Bio-based and biodegradable polymers have been gaining popularity in various sectors and industries for many years. This applies to unfilled materials and composites, as well as mixtures of biodegradable polymers [1,3,4,5,6,7,8]. The production of composites using bio-based and biodegradable polymers follows similar principles to those applied in the processing of petroleum-based polymers. However, combining these two types of polymers—despite their fundamentally different life cycles—is an intriguing approach. While bio-based polymers are designed to break down into simple compounds like H_2_O, CO_2_, and NH_4_, petroleum-based polymers are typically either recycled or used for energy recovery [9,10,11,12].

A crucial parameter for composites made from biodegradable polymers is the type of filler used. It seems reasonable to use mineral fillers with a specific pH (such as talc, chalk) and fillers of plant origin with different shape coefficients (such as fibrous fillers with various lengths and diameters of fibers and/or fillers in the form of wood flour and biochar) [13,14,15,16,17,18,19,20,21,22,23,24,25]. One of the primary objectives in developing and utilizing mixtures of bio-based and biodegradable polymers is to modify the functional characteristics of the final product. This includes enhancing its mechanical properties, such as stiffness, which is particularly relevant in packaging applications, as well as improving its processing behavior, including thermal resistance during manufacturing processes [2].

Luzi et al. [26] analyzed the effect of various types of polymer mixtures (bio-based and fossil-based) and the type of filler on the properties of the obtained products. They focused on the solutions used in the packaging industry. The authors concluded that mixture materials consisting of bio-based polymers, fossil-based polymers, and fillers have extraordinary potential. According to the authors, materials of this type have significant potential for enhancing moisture and gas barrier properties [6,26].

A similar approach was proposed by Culebras et al. [27], who analyzed bio-based, structurally compatible polymer mixtures using lignin and carbon fiber-based precursors. The results showed excellent compatibility between the polymers and fillers (lignin) used. Furthermore, the studies demonstrated that materials of this type have extraordinary potential as precursors for coal production [27].

Further aspects related to the creation of bio-based polymer blends were raised by Makhijani et al. [5], focusing on the biodegradability of such materials. The authors created compositions containing starch, poly(saccharides), cellulose, chitin, PLA, banana fibers, and coffee processing waste. The analysis indicates that various industries are growing interested in materials such as biopolymeric blends. The authors highlighted issues related to the fillers used and their dispersion in the polymer matrix, similar to the case with fossil-based materials [5,28].

Research shows that biodegradable polymers and fillers often require auxiliary agents, known as compatibilizers, to enhance their properties. Imre and Pukánszky explored these aspects [29]. The authors focused on materials such as poly(lactic acid) (PLA), starch, poly(hydroxyalkanoates) (PHA), poly(caprolactone) (PCL), and poly(hydroxybutyrate) (PHB), among others. Their studies showed two primary findings: first, the necessity of using intermediates in the processing of biodegradable polymer mixtures, and second, significant deficiencies in the nomenclature and regulations concerning biodegradable polymers.

The conclusion was that the currently used thermoplastic bio-based polymers require modifiers to adjust their properties for specific applications. The authors suggest that mixing biodegradable polymers is a method for achieving new properties, particularly for aliphatic polyesters [7,29,30]. However, this approach primarily focuses on the creation of mixtures and composites based on bio-based and biodegradable polymers combined with fillers of plant origin. In recent years, scientists have reported an increasing interest in creating intentional mixtures of fossil-based and bio-based polymers, which have been used for many years.

These mixtures are intentionally created, resulting in innovative material solutions that combine the features of materials derived from crude oil with those of bio-based materials [31]. The discussion should begin with obtaining such mixtures [32,33]. It should be noted, however, that researchers’ efforts are mainly focused on materials belonging to the group of polyolefins (PE, PP) and (PLA) (regarding biodegradable polymers) [34,35]. Most studies concentrate on obtaining mixtures of petroleum-derived polymers with biodegradable polymers while maintaining unchanged or slightly modified mechanical, rheological, or thermal properties [36,37,38,39].

Moreover, mixtures of this type have also become a matrix for composites, which, according to assumptions, should improve the properties of the obtained materials and reduce the consumption of individual polymer components [40,41,42,43,44,45,46,47].

The research aimed not to create new polymer blends but to verify the properties of the resulting polymer mixtures that can be made through an uncontrolled and unsupervised process of recycling, mixing, and then recycling waste from bio-based and petroleum-based polymers. The authors aimed to draw the attention of a broader audience to the growing share of bio-based and biodegradable polymers in the European and global markets. These polymers, which visually and organoleptically do not differ from petroleum-based polymers, can be incorporated into the general waste stream and processed alongside traditional waste.

The work is a simulation of a situation that may arise in the mechanical recycling of thermoplastic polymers and is a response to the concerns of the recycling industry, which is beginning to grapple with the appearance of biodegradable plastics in the waste stream. This publication aims to analyze and verify the unintended incorporation of varying amounts (mass content ranging from 1 to 10 wt.%) of biodegradable and bio-based polymers into petroleum-derived plastics. The study examines the mechanical and thermomechanical properties of the resulting mixtures, aiming to establish a correlation between the biopolymer content and observed changes in these properties. Additionally, the research aims to investigate whether the presence of biopolymers affects the structural integrity and processing behavior of the material mixtures. The authors also evaluate the potential impact of these modifications on the recyclability and long-term stability of the materials. Ultimately, the findings offer valuable insights into the feasibility of integrating bio-based components into conventional polymer matrices without compromising their essential performance characteristics.

## 2. Materials and Methods

### 2.1. Tested Materials

Three types of thermoplastic polymer materials: (1) polypropylene (PP), (2) polystyrene (PS) and (3) a mixture of poly(lactic acid) and poly(butylene adipate terephthalate) (PLA/PBAT) called in the next part of manuscript as (BIO) and their blends were used in the conducted investigations.

Polypropylene (PP) was selected as the first material for this study, specifically Moplen HP500N virgin polypropylene, supplied by Basell Orlen Polyolefins (Plock, Poland). As a thermoplastic, partially crystalline polymer belonging to the polyolefin group, polypropylene is widely used in the packaging industry due to its favorable mechanical properties and processability.

Polystyrene (PS) is an amorphous material and was selected due to its popularity in numerous applications in everyday life. The polystyrene used in this study, Styrolution (PS) 165N/L, was manufactured by INEOS Styrolution Group GmbH (Frankfurt, Germany) and was selected. Material (PS) 165N/L is a general-purpose (PS) manufactured by INEOS, characterized by its high rigidity and good dimensional stability. Additionally, this material offers favorable mechanical properties, including impact resistance and thermal stability, which contribute to its widespread use in packaging, consumer goods, and industrial applications.

The biodegradable polymer selected for the study was a mixture of poly(lactic acid) (PLA) and poly(butylene adipate terephthalate) (PBAT) AnBioS3, manufactured by AFC Ecoplastics (Costa Mesa, CA, USA). This biodegradable polymer is primarily designed for extrusion and is referred to in the manuscript as (BIO) polymer.

The main objective of the research was to analyze the impact of mixing fossil-derived thermoplastic polymers, which are the main components of the post-consumer waste stream (PCR), particularly food packaging with a very short product life cycle, with bio-based and biodegradable polymers, which are increasingly used for the production of packaging, among other applications.

The next step was to prepare polymer blends after selecting the virgin materials/raw materials. A biodegradable (BIO) polymer was added to polypropylene and polystyrene in different weight ratios of 1; 5 and 10 wt.% (Table 1). The blends were not filled with any filler. The amount of biodegradable polymer (BIO) was selected based on the authors’ experience and available knowledge. In the case of intentionally produced blends, the ratio of the share of individual components depends on the desired properties, such as stiffness; hence, mass shares of, for example, 80–20, 70–30 are preferred. To better reflect the real situation that can be observed during the reprocessing of these two material groups and based on statistical data [2], which indicates a still small share of bio-based polymers in the market, the authors stated that the percentage share of the biodegradable polymer should be equal to or below 10 wt.%.

**Figure 1:**
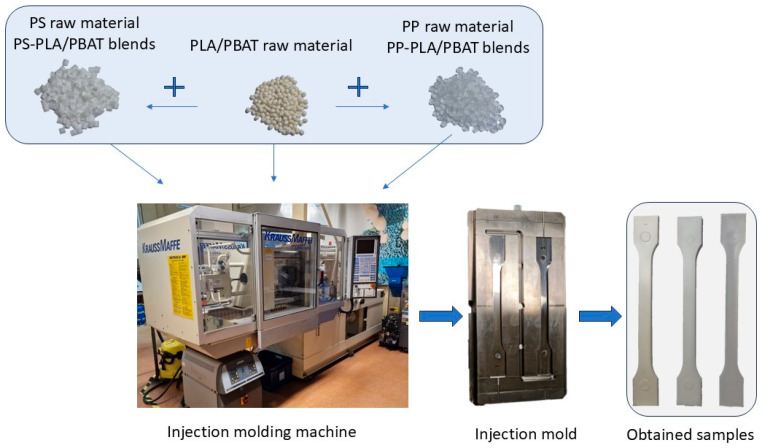
Schematic diagram of the process of preparing polymer blends to obtain standardized samples.

The Krauss Maffei KM65 (Munich, Germany), a hydraulic injection molding machine equipped with a two-cavity insert mold and thermostated with a temperature control system, was used to produce standardized specimens in accordance with PN-EN ISO 294-1 [48] and PN-EN ISO 527-1 [49] standards. The homogenization and mixing process was conducted in the plasticizing unit of the injection molding machine, not in the sense of industrial processes of compounding using twin-screw extruders. Schematic diagram of the process of preparing polymer blends to obtain standardized specimens shown in Figure 1.

The mass of each polymer, in the form of a masterbatch, was precisely measured using a laboratory scale, and then the polymers were mechanically mixed. Further mixing was carried out in the plasticizing system of the injection molding machine by selecting optimal processing parameters, including plasticizing time, rotational speed, and injection cylinder temperature.

### 2.2. Technologies Used to Manufacture Granulates and Standardized Samples

The parameters related to the preparation of individual materials for the technological process (drying time and drying temperature) and the parameters of the injection process of individual base materials (PP, PS, BIO PLA/PBAT) were selected in accordance with the information contained in the Technical Data Sheets (TDS) technology cards. The hot air drying machine type CD-9 produced by SHINI Europe, Italy, was used in raw materials preparation.

As the data in the studies show (Table 2), petroleum polymers are not highly hygroscopic materials. For (PP), drying is not required at all, while for (PS), only preliminary drying is needed to remove condensed moisture. On the other hand, (BIO) requires a long-term process of removing moisture, indicating its high hygroscopicity and ability to absorb moisture.

For the base materials, the parameters were selected in accordance with the principles of plastics processing and the guidelines included in the safety data sheets provided by the manufacturers (Table 3).

However, the injection parameters were modified for mixtures obtained from both base materials (PP and PS), primarily concerning the temperature of the injected plastics. Due to the increasing content of biodegradable polymer, the injection temperature of (PP) was reduced from 230 °C to 210 °C, and that of (PS) from 210 °C to 200 °C. This methodology was introduced to avoid the degradation of the processed mixtures. Biodegradable polymers are sensitive to thermal loads, and exceeding the recommended thermal ranges can lead to rapid thermal decomposition.

The processing parameters used during the processing of individual raw materials (virgin) and polymer blends were selected based on the analysis of the technical data sheets for the processed materials. The parameters changed slightly for the processed blends, especially for the mixtures containing 10% biodegradable polymer. For the processed mixtures, the injection temperature was reduced to prevent degradation of the biodegradable fraction in the mixture.

Standardized samples for raw materials and polymer blends were manufactured using injection molding technology. The samples were manufactured in accordance with PN-EN ISO 294-1 [48] and PN-EN ISO 527-1 [49] standards, as type 1A test samples with a length of 150 mm and a cross-section of 10 mm × 4 mm. The distance between the grips in the uniaxial tensile test was 115 mm, following the parameters presented in Table 3. The constant parameters for all investigated materials were: mould temperature (40 °C), injection pressure (80 MPa), and holding pressure (65 MPa).

Thirty pieces of the type 1A samples of each mixture were made during injection molding, and most tests were conducted on five samples and presented as a mean value with standard deviation. DMTA tests, as well as hardness, impact strength, and tensile strength measurements, were performed using the standardized specimens obtained.

### 2.3. Measurements of the Density of the Raw Polymers and the Obtained Blends

As part of the conducted research, new blends were produced. Therefore, one of the basic studies measured the density of the input materials (base polymers) and the obtained materials. This aspect is crucial, as each tested material exhibits a distinct density value. Therefore, it is essential to examine how an increasing proportion (percentage content) of the biodegradable polymer influences the fundamental physical properties of petroleum-derived polymers, which serve as the primary component of the composition. The density of the raw materials and the obtained mixtures was measured. Samples for density measurement were taken from standardized moldings (the density of the granulates was not analyzed). Density measurement was carried out in accordance with PN-EN ISO 1183-1 [50] standard. The density values of the base materials were taken from the TDS cards and are presented in Table 4.

Density measurement was performed using immersion on a measurement station consisting of a Radwag AS 220.3Y laboratory balance (Radom, Poland) with auxiliary equipment. Density was measured in 5 replications.

### 2.4. Analysis of Mechanical Properties—Tensile Test

Standardized type 1A specimens were prepared using thermoplastic injection molding technology to compare the static mechanical properties. The manufacturing process adhered to the parameters specified in Section 2.2, with careful consideration of the specific drying requirements of each polymer, including drying time and temperature. The preparation of polymer mixtures followed the same methodology.

All specimens complied with PN-EN ISO 294-1 [48], ensuring consistent sample and beam geometries. The mechanical property analysis was conducted in accordance with PN-EN ISO 527-1 [49], providing a systematic evaluation of the materials’ performance characteristics.

After the injection, the samples were subjected to a conditioning process for 24 h at a temperature of 23 °C and a relative humidity of 50%.

The standardized samples prepared in this manner were subjected to a statistical tensile strength test at a speed of 50 mm/min. Five replications were performed for each type of material. The following values were determined during the measurement: tensile strength and elongation at break. A universal testing machine from Hegewald & Peschke Meß- und Prüftechnik GmbH (Nossen, Germany) equipped with a 20 kN force sensor was used to determine these values.

Tests for mechanical properties were conducted at a temperature of 22 °C. These tests were conducted to determine the effect of additives/contaminants—in this case, the introduction of a biodegradable polymer added in various mass ratios—on the mechanical properties of products processed using injection molding technology. These tests were conducted to assess the variations in mechanical properties that may arise in thermoplastic polymers due to the uncontrolled dosing of bio-based polymers into the petroleum-derived waste stream, particularly during the mechanical recycling process.

### 2.5. DMA—Dynamic Mechanical Analysis of the Obtained Blends—Test Procedure

Since polymer mixtures composed of petroleum-based and biobased components remain insufficiently studied and understood, the authors designed an experiment to comprehensively analyze their thermomechanical properties. This investigation employs dynamic mechanical thermal analysis (DMTA) methodology to provide insights into the structural and performance characteristics of these material mixtures. These studies were carried out in accordance with PN-EN ISO 6721-1 [51].

A measuring device from NETZSCH—the DMA 303 Eplexor (Selb, Germany)—was used to perform thermomechanical tests accurately. The tests employed the three-point bending method without restraint. The measurement was performed within a temperature range of −150 °C to +100 °C, utilizing supports positioned at a fixed distance of 50 mm. All obtained data were evaluated in the dedicated software Proteus 9.2.

### 2.6. DSC—Differential Scanning Analysis—Test Procedure and Equipment

The analysis of the structure properties was carried out by differential scanning calorimetry. The samples were cut off from the middle part of the injected samples. The main aim of the DSC test is to obtain data and information about the effect of the addition of (BIO) to (PP) and (PS) on the changes in thermogram flow and values of characteristic temperatures of transitions (melt peak, crystallization peak, and their range). These studies were conducted in accordance with PN-EN ISO 11357-1 [52].

The measurements were performed using the DSC Polyma 214 device, produced by Netzsch (Selb, Germany). The temperature range was RT up to 180 °C with a heating rate of 10 K/min. The average mass of the sample was approximately 12 mg. The H-C-H (1st heating—1st cooling—2nd heating) program was employed in the investigations; however, only the 1st cooling—2nd heating curves were utilized in the evaluation due to stress relaxation during the slow cooling stage (10 K/min). All obtained data were evaluated in the dedicated software Proteus 9.2.

### 2.7. Charpy Impact Strength Analysis of Standardized Test Samples Obtained from Raw Materials and Their Blends

The conducted work and further analysis of the mechanical properties of raw materials and obtained blends also included verification of impact strength. Impact strength measurements were performed in accordance with PN-EN ISO 179-1 [53] with the use of the impact tester Zwick/Roell Hit 5.5 P device (Ulm, Germany).

Prior to the measurement, the samples were conditioned for 24 h at a temperature of 23 °C and a relative humidity of 50%. Standardized specimens were used in the impact strength measurements. The samples with a Type A notch of 2 mm depth, an angle of 45°, and a rounding radius of 0.25 mm were tested. The notches were cut using a Zwick/Roell notch cutter type 077097 (Ulm, Germany). After the notch was cut, the specimens were placed on the measuring supports (with a 62 mm distance between the supports), and then the hammer lock was released. The measurement was performed under controlled conditions at a temperature of 23 °C and a relative humidity of 50%. A pendulum/hammer with an energy of 0.5 J was used in the measurements. Impact strength measurements were conducted to assess whether the incorporation/dosing of the bio-based polymer (BIO) into polypropylene (PP) and polystyrene (PS) influences their behavior under dynamic loading conditions.

### 2.8. Analysis of Changes in Hardness of Standardized Samples Using the HRM Method

The processing of polymer blends can also lead to changes in the fundamental physical properties of thermoplastic plastics. Hence, hardness measurements were also carried out using the Rockwell method. The tests were conducted in accordance with PN-EN ISO 2039-1 [54] based on the M (HRM) method. A Sinwon DigiRock DP3 (Dongguan, China) hardness tester was employed. Following the standard guidelines, a steel ball with a diameter of 6.35 mm was used. The hardness measurement was divided into two stages. The first stage involved applying a pre-load force of 10 N, and the second stage involved applying a target force of 980 N. The force application time was 15 s. The hardness test was performed on three samples of each raw material and blend, with three replications for each sample.

### 2.9. The Microscopic Examination of Sample Fractures

The microscopic observations were conducted as a complement to aid in the analysis of all the tests performed in the experiment. The digital microscope VHX-900F, equipped with a graphics processor and a motorized *Z*-axis, produced by Keyence (Osaka, Japan), was used in these investigations.

## 3. Results and Discussion

### 3.1. The Results of the Analysis of the Density Measurement of the Tested Materials

Density measurements were performed on samples taken from standardized moldings obtained during the injection molding process. Samples were taken by mechanical cutting, using a blade to get smooth, unfrayed edges. The measurement itself was performed using the immersion method. The data obtained for both raw materials and the obtained blends are presented in Table 5.

According to the obtained data, the (BIO) showed the highest value of density, whereas polypropylene showed the lowest. However, it should be noted that with the increased amount of biodegradable polymer (BIO) in the mixture, the density of the polymer mixtures also increases. A 3% increase in density was observed for both polypropylene and polystyrene following the addition of 10% (BIO) bio-based polymer. This consistent change highlights the impact of biopolymer incorporation on the material’s structural properties.

As studies have shown, the uncontrolled mixing of biobased biodegradable materials in municipal waste (primarily based on polyolefins) can impact physical properties, leading to, among other things, an increase in density.

### 3.2. The Results of the Analysis of the Mechanical Properties of the Tested Samples

The static tensile strength test was conducted for the raw materials and the obtained mixtures. Five repetitions were performed for each type of material. From the series of obtained values, two representative values were presented, relating to the tensile strength, expressed in MPa, and the strain at break of the sample, expressed as a percentage. The comparison of these two values is presented in Table 6.

Mechanical property tests conducted in accordance with the PN-EN ISO 527-1 [49] static tensile strength method demonstrated that the formation of polymer mixtures from both amorphous and semi-crystalline materials results in distinctly different behavior of standardized specimens. As a material, (BIO) is characterized by the highest tensile strength value (over 30 MPa) and the lowest deformation value (4.83%). The tensile strength of polypropylene was slightly lower, amounting to 28.7 MPa, while for (PS), it was 13.57 MPa. However, it should be noted that the obtained deformation of (PP) was at the level of 134%, while for (PS), it was 51%.

The creation of mixtures with the content of biodegradable polymer PLA/PBAT in the amounts of 1%, 5%, and 10% introduced significant changes in the obtained values. Analyzing the changes in behavior and obtained values, it should be noted that for (PP), 1% (BIO) content causes an increase in tensile strength by 3.37% (to 29.67 MPa). However, the 1% content of the (BIO) fraction in (PP) causes deterioration in its deformability, with a recorded decrease of 3.97% (to 129.11%). Further increases in (BIO) content in (PP) cause a simultaneous reduction in both tensile strength and deformability. While the tensile strength value decreases by 4.35% for the (BIO) content of 10% in the mixture, the deformation value at 10% (BIO) content decreases by 90.61%.

Completely different behavior was recorded for (PS). With the increase in biodegradable polymer content in the mixture, both tensile strength and deformation values increased. The highest increase in tensile strength was observed for the 10% (BIO) content, which increased by 37.73%. In the case of deformation, the highest growth was recorded for the 5% (BIO) treatment, which was equal to 32.55%.

As studies have shown, the reaction or behavior of materials depends on the material group (amorphous or semi-crystalline materials), exhibiting different mechanisms. Mixing (PP) with a (BIO) (biobased and biodegradable polymers) results in a slight decrease in mechanical properties, defined by tensile strength, and a radical reduction in deformation (strain). For finished products, in the case of creating such a mixture (whether in a recycling plant or at the processor), it will result in a significant reduction in the possibility of deformation under external loads and will preserve the detail. Therefore, this effect should be included among the adverse effects of blend preparation [7,37].

A different behavior was observed for the amorphous polymer (PS). With the increasing ratio of (BIO) content in (PS), an increase in both tensile strength and strain values was observed. However, it should be noted that the use of such mixtures, despite obtaining encouraging results, should not be advisable because both polymers belong to different material groups. Further analysis of the properties of such mixtures and the long-term effects of creating this connection is necessary, including microscopic examinations (both optical and SEM) and aging studies.

### 3.3. The Results of Dynamic Mechanical Analysis of the Raw Materials and Obtained Blends

To perform the dynamic mechanical analysis, the DMA 303 Eplexor analyzer (Netzsch, Germany) was used. The test samples were taken from the injection moldings and cut to the sample holder supports dimension (50 mm). A deformation of 120 μm with a frequency of 1 Hz was used during measurements. The heating rate increased at 3 K/min in the range from −150 °C to 100 °C.

**Figure 2:**
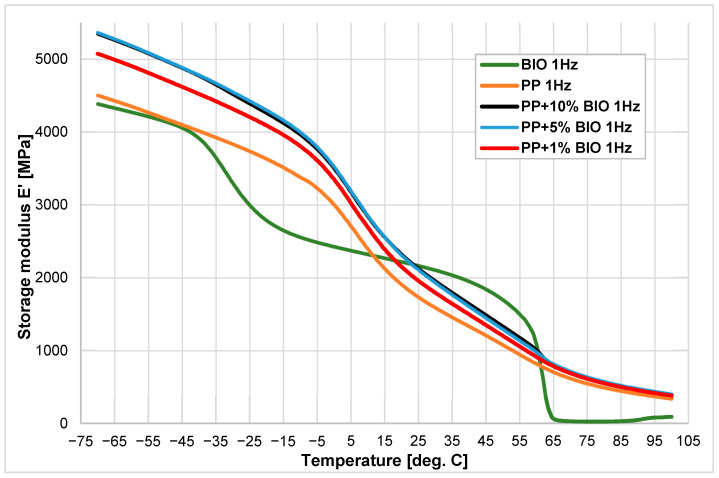
Storage modulus E’ vs. temperature of (PP), (BIO), and its blends with concentrations of 1, 5, and 10% of (BIO).

**Figure 3:**
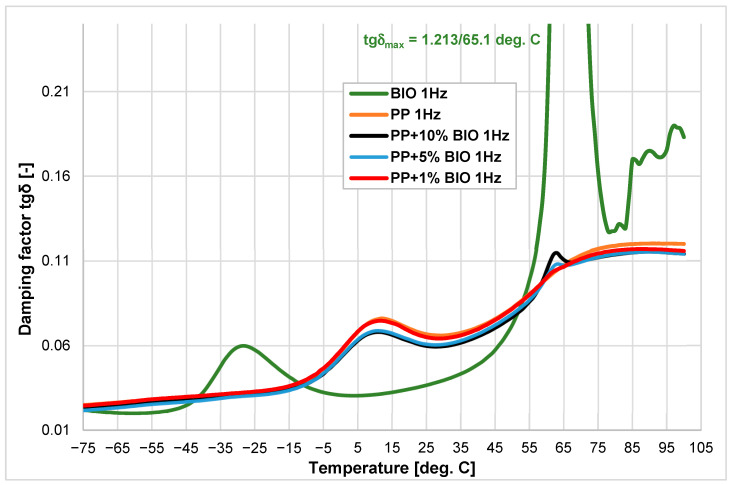
Damping factor tgδ vs. temperature of (PP), (BIO) and their blends with the addition of 1, 5, and 10% of (BIO).

**Figure 4:**
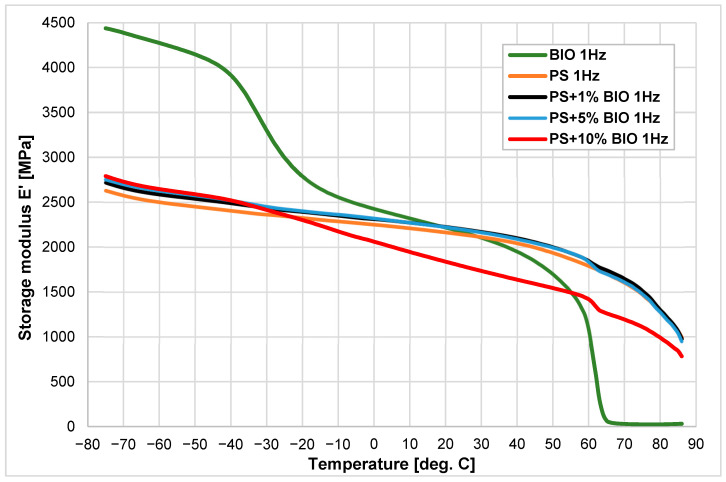
Storage modulus E’ vs. temperature of (PS), (BIO) and their blends with the addition of 1, 5, and 10% of (BIO).

**Figure 5:**
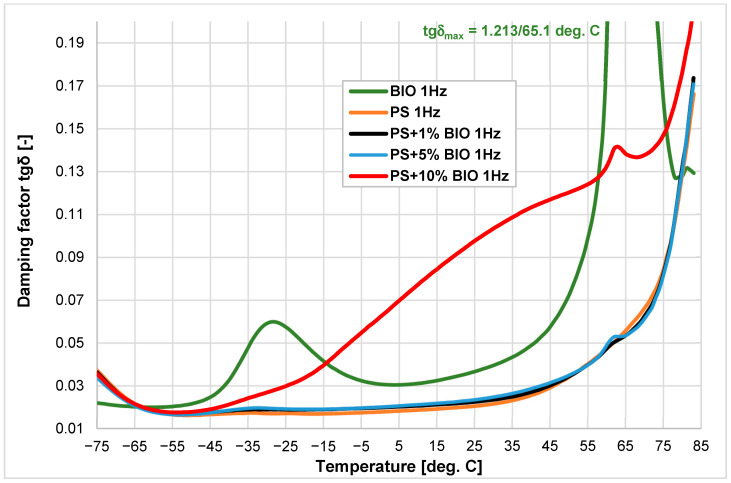
Damping factor tgδ vs. temperature of (PS), (BIO) and their blends with the addition of 1, 5, and 10% of (BIO).

The changes in the elastic storage modulus (E’) and mechanical loss factor (tgδ) vs. temperature were recorded. The thermograms were presented in Figure 2, Figure 3, Figure 4 and Figure 5. The first two figures (Figure 2 and Figure 3) present E’ and tgδ for different concentrations of the biopolymer (BIO) in (PP) with 0%, 1%, 5%, and 10% of the biopolymer. The following figures (Figure 3 and Figure 4) illustrate identical concentrations of the (BIO) mixed with the (PS). The green and red curves present the storage modulus (E’) versus temperature of virgin materials used in the experiments. The biopolymer exhibits two phase transitions at −40.1 °C and 58.3 °C. For (PP), only one onset was recorded at −5.2 °C. The storage modulus (E’) for the biopolymer increases over the value of (PP) in the temperature range of 20–60 °C. Adding just 1% (BIO) to (PP) results in a significant increase in the storage modulus of approximately 10%. This does not correspond with the expected results, as the addition of (BIO) to (PP) should decrease stiffness, especially at temperatures below 10 °C. This may indicate that the (BIO) additive causes such an arrangement in the (PP) polymer matrix that increases the storage modulus (Figure 2).

The change in tgδ is presented in Figure 3. The biopolymer shows a very high peak of tgδ at 65.1 °C (corresponding with (PLA) glass transitions), and its effect is only notable in the 5% and 10% mixtures. The glass transition temperature of all mixtures is similar to the value of (PP), ranging between 10.6 °C and 12.0 °C. Analyzing the tgδ course for the (BIO), it can be stated that despite the significant values of this parameter at temperatures of −28 °C (for the (PBAT) component of (BIO) material) and 65 °C (up to 1.221 for (PLA) component), its influence at these temperatures becomes visible only above 5% content. At the same time, the addition of 1% of the (BIO) fraction causes a decrease in the (PP) base damping properties as indicated by the tgδ loss factor.

The results of the content of (BIO) in (PS) mixtures look different compared to (PP) (Figure 4). The addition of 10% biopolymer significantly decreases the storage modulus to the same range as (BIO). The onsets have very similar values: −35.8 °C (BIO) has −40.9 °C and the second at 60.8 °C (BIO has 48.7 °C). This shows that the addition of biopolymer to amorphous thermoplastic polymers at 1–5% does not change the properties significantly, but above 5% there are substantial changes, especially above the glass transition temperature of the biomaterial (−40 °C to +50 °C).

This substantial effect is also reflected in the tgδ factor. A significant deviation in the tgδ curve value from the original is observed only at a 10% addition of (BIO), whereas the variations remain minor at 1% and 5% concentrations.

The addition of (BIO) to (PS) causes the stiffness (as indicated by the storage modulus E’) to be maintained, especially above the temperature of −20 °C (Figure 4). In contrast to the storage modulus, the mechanical loss factor tgδ (the damping coefficient) gains a significant value above −20 °C due to the very high tgδ value for (BIO). However, these effects are only visible at the content of 10% (BIO) (Figure 5).

During the mixing of biopolymer with semi-crystalline polymer (polypropylene and amorphous (PS), better results from a practical point of view were obtained for (PP). Even the addition of 1% (BIO) resulted in a visible increase in storage modulus by about 7–9%. Further addition of biopolymer, above 5–10%, did not result in such a significant change. In the case of polystyrene (PS), only the addition of 10% of (BIO) caused a substantial decrease in the storage modulus above the temperature of −20 °C. The obtained results indicate that for (PS), only a biopolymer contamination content above 5% will cause visible differences in the stiffness of the injected parts.

These obtained results are significant from a practical point of view because they demonstrate the differential impact of different contents of biopolymer on amorphous and semi-crystalline materials.

### 3.4. The Results of Differential Scanning Calorimetry

**Figure 6:**
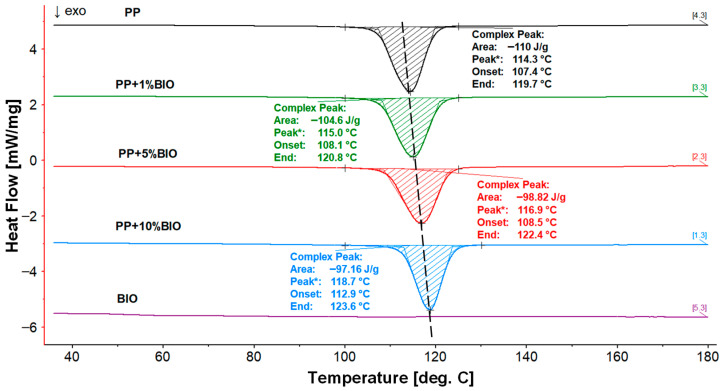
DSC thermograms of the crystallization process during the 1st cooling of (PP) and its blends, * maximum value of peak crystallization.

**Figure 7:**
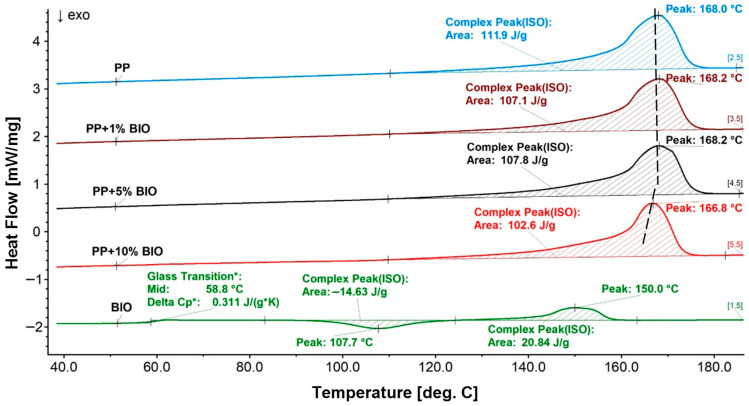
DSC thermograms of melting during the 2nd heating of (PP) and its blends. * values of Glass Transition.

**Figure 8:**
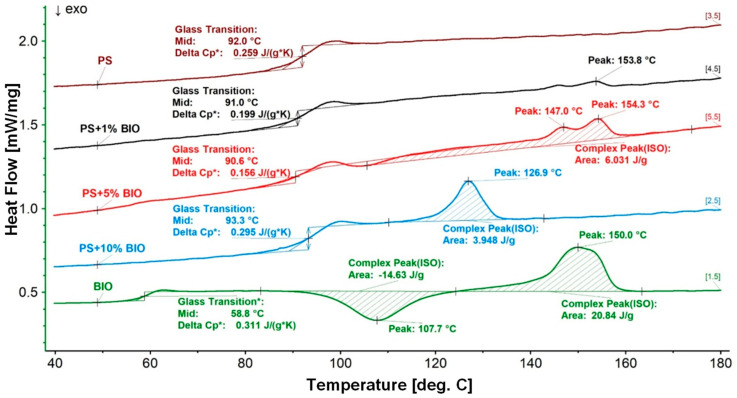
DSC thermograms of melting during the 2nd heating of (PS) and its blends. * value of Glass Transition.

The H-C-H (1st heating–cooling–2nd heating) program was used in the investigations, and the results are presented in the summary charts for all samples in the group of main polymers (PP) and (PS) (Figure 6, Figure 7 and Figure 8). The second heating curves and the cooling phase during which crystallization occurs were selected for analysis. The direction of the exothermic peak is marked on the graphs with an upward arrow. The results were presented in Figure 6 and Figure 7 for (PP) + (BIO) in different contents of (BIO) and in Figure 8 for (PS) + (BIO).

It can be observed that even a slight addition of 1% (BIO) to (PP) initiates changes in the crystallization transformation process. The crystallization temperature showed a shift towards higher values, reaching 118 °C with a 10% addition of (BIO); simultaneously, the crystallization enthalpy decreases to −95.62 J/g. In comparison, pure (PP) shows a crystallization temperature of 114.3 °C and an enthalpy of −110.4 J/g. These changes indicate the influence of (BIO) incorporation on the thermal behavior of (PP). One can find a similar effect of the (BIO) addition to pronucleating agents, which causes a similar effect of shifting the crystallization towards higher temperature values. However, the addition of (BIO) did not cause a significant change in the melting transformation of (PP). The melting temperature of all samples is around 168 °C with a decreasing melting enthalpy from 114.7 J/g for (PP) to 104.6 J/g for the mixture of (PP) with the addition of 10% (BIO).

Figure 8 presents a summary of all (PS) and (BIO) blends and raw materials (PP and PS) used in the tests. Polystyrene is characterized only by a glass transition temperature of 92.9 °C. In comparison, the (BIO) material shows a low glass transition temperature of 58.8 °C during the second heating, which corresponds precisely to the result obtained in the DMTA tests. Further heating of the material shows crystallization before melting (cold crystallization) at 107.7 °C and a sharp melting peak at 150 °C.

The prepared blends exhibited small melting peaks at 1%, 5%, and 10% (BIO) in the (PS) matrix. However, these endothermic effects—observed at 153.8 °C for (PS) + 1% (BIO), 154.3 °C for (PS) + 5% (BIO), and 126.9 °C for (PS) + 10% (BIO)—did not lead to an improvement in the material’s properties.

### 3.5. Impact Strength Analysis of Raw Materials and Obtained Blends

As part of further studies, tests were undertaken to verify the impact strength of both base materials and the obtained blends. Five replications were performed for each material, and the obtained values are presented in Table 7.

The obtained impact strength test results demonstrate consistency. The tests showed that the biodegradable material (BIO) is characterized by a low impact strength value (2.81 kJ/m^2^), and its addition to PP and (PS) results in a decrease in the impact strength of both material groups. However, some exceptions to this rule should be noted.

For (PP) mixtures with the content of (BIO) in the amounts of 1% and 5%, the decrease in the impact strength value was recorded (deterioration of this value by 20%). On the other hand, for the mixture of (PP) with 10% content of (BIO), a 7.74% increase in the impact strength value was observed.

For polystyrene, the mixture containing 1% (BIO) showed an increase in impact strength of 13.8%. A further rise in the biodegradable polymer content results in a 24.44% decrease in the impact strength value for a blend containing 10% (BIO).

The obtained results indicate that the mixing of biodegradable polymers with amorphous or semi-crystalline polymers can cause unpredictable alterations in their properties.

### 3.6. The Results of the Analysis of Hardness Standardized Parts According to the Rockwell Method M

As in previous studies, it is crucial to analyze and determine the changes in the hardness values of the base materials, as well as those of the obtained mixtures. For this purpose, the Rockwell hardness measurement method and the M method, as specified for polymers, were used. The tested materials belong to the group of thermoplastic polymers; therefore, a steel ball with a diameter of 6.35 mm and a load value of 980 N was used in the tests. The test was conducted on three samples of each material. The obtained results are presented in Table 8.

Hardness results using the Rockwell M method (Table 8) showed that adding the (BIO) polypropylene and polystyrene materials causes a decrease in the hardness value. For polypropylene, the 10% addition of a (BIO) led to an 8% decrease in the hardness value. For (PP), this property deteriorated by 9.78% for the mixture with the 5% addition of (BIO). The addition of 10% (BIO) to (PS) results in a slight increase in the hardness value. Still, it remains lower than the initial hardness value of (PS) without filling.

The measurements showed that the hardness of the biodegradable polymer is 14.15 on the HRM scale. Hence, the decrease in the hardness value of both material groups after the production of mixtures may be caused by the weakening of the structure of the base material. The addition of biodegradable polymer into polypropylene or polystyrene results in local structural weakening and a reduction in penetration resistance. Consequently, an increasing proportion of the biopolymer fraction is expected to further compromise the integrity of both material groups, leading to a progressive decline in hardness.

### 3.7. The Results of Microscopic Observations of the Obtained Samples

**Figure 9:**
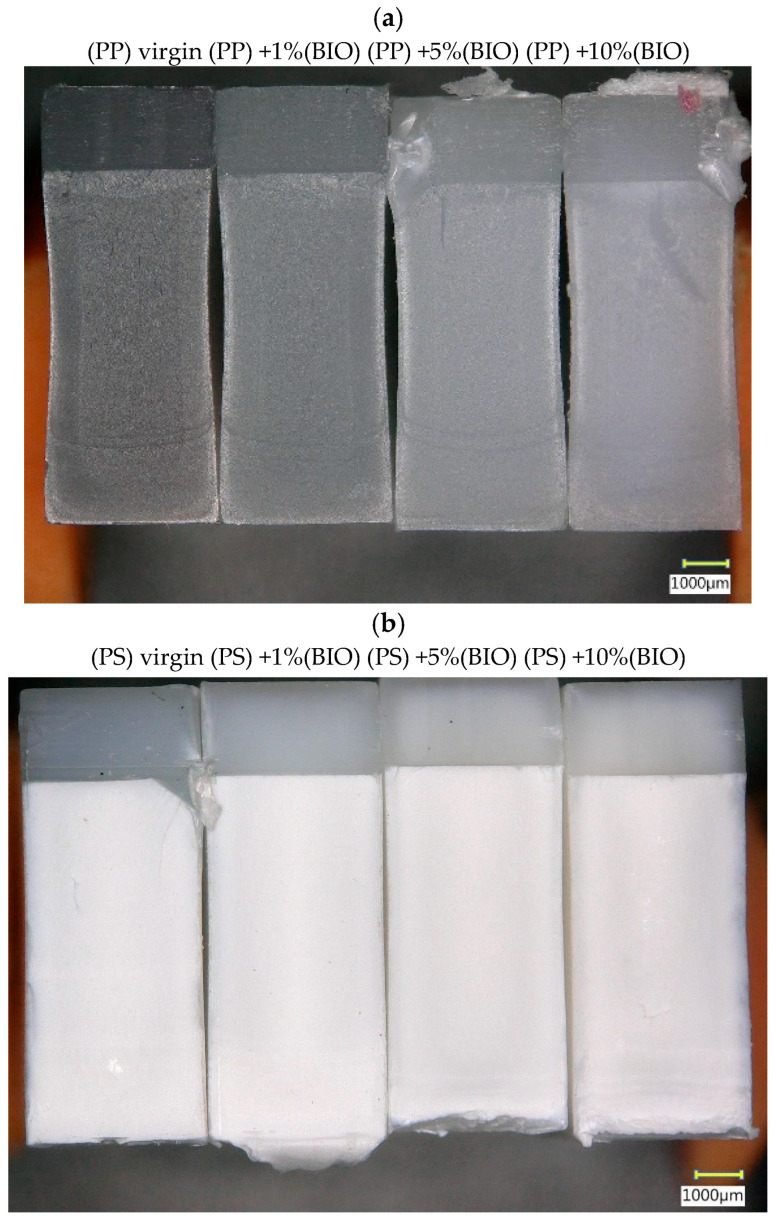
Microscopic view of samples after impact strength tests.

Figure 9a,b show microscopic images of sample fractures from Charpy impact tests. A trace of the notch cut is visible in the upper part of the sample. Crack propagation during the Charpy test starts from the edge of the notch and proceeds towards the lower part of the sample.

For the sample of the (PP) mixtures, it can be observed that even a 1% addition of (BIO) causes a visible lightening of the sample color. For additions of 1% and 5%, the material is evenly distributed throughout the sample mass. Only in the case of a 10% (BIO) addition, a certain swirl or a small area of lack of homogeneity can be observed in the upper part of the sample, just below the notch. For the (PS) mixtures, the samples are even in appearance. However, the addition of 1% (BIO) caused hinge (elastomeric) cracking of the samples, and, similar to the (PP) mixtures, the addition of 10% caused some disturbances in the cracking process.

## 4. Conclusions

As part of the work, it was assumed that the properties of the input materials would be analyzed, and polymer mixtures would be created with fillings of 1%, 5%, and 10%. According to these assumptions, polypropylene (Moplen 500N) and polystyrene (Styrolution (PS) 165N/L), which are among the most popular partially crystalline and amorphous materials, were doped with a bio-based polymer (BIO) in the injection molding process.

Impact of (BIO) contamination on density—Even small additions (1–10 wt.%) of (BIO) to polypropylene (PP) and polystyrene (PS) lead to a measurable increase in density, which may be critical for quality control during recycling. A consistent rise of approx. A 3% increase was observed at a 10% (BIO) content.

In the field of mechanical properties, different responses of semi-crystalline and amorphous matrices were obtained. In (PP), (BIO) contamination reduced deformability drastically (up to 90% at 10% BIO) and slightly lowered tensile strength. This indicates that even minor mixing with biopolymers can compromise the ductility and serviceability of semi-crystalline (PP). In (PS), a contrary effect was recorded: (BIO) additions increased both tensile strength and elongation at break, especially at 5–10%. This suggests limited compatibility in amorphous matrices but also highlights unpredictable property changes.

Thermal analysis (DMA, DSC)—for PP, the addition of 1% (BIO) unexpectedly increased the storage modulus (stiffness) by 7–9%. (BIO) acted similarly to a nucleating agent, shifting crystallization temperatures upward. For (PS), property changes became significant only at ≥10% (BIO), where stiffness decreased while damping factor values increased. This indicates that low-level contamination may remain undetected, but higher contents substantially alter behavior.

Impact strength and hardness—BIO additions generally reduced impact resistance and hardness in both (PP) and (PS). Only isolated cases (e.g., (PP) with 10% (BIO)) showed slight improvements, which were not consistent across blends. Overall, the incorporation of BIO weakens structural integrity.

Microstructural observations—Microscopy confirmed that low (BIO) levels distribute homogeneously, while higher fractions (10%) introduced visible inhomogeneities, potentially explaining property deterioration.

Recycling implications—The study demonstrates that the uncontrolled contamination of fossil-derived plastics with biodegradable polymers can unpredictably alter their mechanical, thermal, and structural properties. In semi-crystalline (PP), adverse effects dominate (loss of ductility, reduced hardness), while in amorphous (PS), mixed but still destabilizing effects occur.

Practical relevance—These findings underline the risk of mixing biodegradable packaging with conventional plastics in municipal waste streams. Even at low contents (<10 wt.%), changes are sufficient to impair recyclate quality. This calls for:

- stricter separation of waste streams,
- better labeling and identification of biodegradable packaging,
- and further studies on compatibilization strategies to minimize property losses.

The conducted tests on mechanical properties (static and dynamic) and thermomechanical properties have shown that the change in the properties of the mixture depends not only on the amount of biodegradable polymer but also on the nature of the load. It would be advisable to analyze mechanical properties in relation to the duration of the load; therefore, a long-term load analysis is necessary. For dynamic tests, a decrease in impact strength was demonstrated with increasing biodegradable polymer content in the produced mixtures. Similar behavior was recorded for hardness measurements.

The research conducted reveals a new and emerging issue: the growing presence of larger streams of biodegradable plastics in municipal waste, as well as potential threats related to the ineffective separation of these plastics during recycling processes. Further research will focus on biodegradability testing under various conditions and expanding the scope of the study to include application areas in the recycling and waste industries.

## Figures and Tables

**Table 1:** Formulation of the selected materials and blends.

Sample No.	Material Code	Original Material Content [%, by Weight]	(BIO) Content [%, by Weight]
1.	PP	100	0
2.	PS	100	0
3.	PLA/PBAT	100	0
4.	PP/PLA/PBAT-1	99	1
5.	PP/PLA/PBAT-5	95	5
6.	PP/PLA/PBAT-10	90	10
7.	PS/PLA/PBAT-1	99	1
8.	PS/PLA/PBAT-5	95	5
9.	PS/PLA/PBAT-10	90	10

**Table 2:** Drying parameters of processed materials.

Material	Drying Temperature [°C]	Drying Time [h]
Polypropylene (PP)	-	-
Polystyrene (PS)	60	2
BIO (PLA/PBAT)	70	5

**Table 3:** Processing parameters of processed materials/blends.

Processing Parameter	PP	PS	BIO	PP/BIO 1–10%	PS/BIO 1–10%
Injection temp. [°C]	230	210	200	210	200
Mold temp. [°C]	40	40	40	40	40
Specific injection pressure [MPa]	80	80	80	80	80
Holding pressure [MPa]	65	65	65	65	65

**Table 4:** Reference density of the raw materials (based on Technical Data Sheets).

Quantity	PP	PS	BIO
Density [kg/m^3^]	900	1020	1250

**Table 5:** Results of density measurement of the raw materials and their blends in the shape of molded test samples (Mean and its standard deviation of 5 samples).

	PP	PS	BIO	PP/BIO 1%	PP/BIO 5%	PP/BIO 10%	PS/BIO 1%	PS/BIO 5%	PS/BIO 10%
Density, [kg/m^3^]	900	1030	1290	900	910	930	1030	1040	1050
Stand. Dev.	4	7	3	1	1	1	2	2	1

**Table 6:** Tensile strength and elongation at break of raw materials and blends.

	PP	PS	BIO	PP/BIO 1%	PP/BIO 5%	PP/BIO 10%	PS/BIO 1%	PS/BIO 5%	PS/BIO 10%
Tensile Strength, [MPa]	28.70	13.57	30.45	29.67	28.46	27.45	17.36	17.57	18.69
Stand. Dev.	3.00	4.92	0.25	1.03	0.83	1.83	1.52	1.85	2.62
Elongation at break, [%]	134.46	51.18	4.83	129.11	72.58	12.62	63.64	67.84	64.02
Stand. Dev.	27.16	2.89	0.35	21.90	15.59	12.62	1.37	9.42	5.29

**Table 7:** Impact strength results of raw materials and blends.

	PP	PS	BIO	PP/BIO 1%	PP/BIO 5%	PP/BIO 10%	PS/BIO 1%	PS/BIO 5%	PS/BIO 10%
Impact strength [kJ/m^2^]	3.1	9.86	2.81	2.96	2.47	3.34	11.17	9.4	7.45
Stand. Dev.	0.08	0.31	0.06	0.11	0.42	0.11	0.11	0.29	0.29

**Table 8:** The results of Rockwell hardness tests HRM.

	PP	PS	BIO	PP/BIO 1%	PP/BIO 5%	PP/BIO 10%	PS/BIO 1%	PS/BIO 5%	PS/BIO 10%
HRM	66.31	39.76	14.15	66.37	64.2	60.96	37.93	35.87	36.46
Stand. Dev.	0.57	1.00	0.57	0.61	1.3	1.18	0.83	1.49	0.72

## Data Availability

The original contributions presented in this study are included in the article. Further inquiries can be directed to the corresponding authors.
